# Tuberculous Leptomeningitis Basilar

**Published:** 1897-10

**Authors:** Gerosa Giuseppe


Tuberculous Leptomeningitis Basilar. By Dr. Gerosa Giuseppe.—In August of last year Giuseppe was called to an ox, two and one-half years old. Although the animal always had a good appetite, and there was no falling off in its general condition, for a month it had been holding its head and neck slightly bowed and turned toward the right. On the day before Giuseppe was called in the ox had suddenly fallen down in the stall. It worked itself up quickly again, but was seized with great restlessness, with which chronic contraction of the muscles of the neck was connected; the animal now took no food. The next day the animal showed a peculiar inclination to press itself against the manger for support. During the examination it retained the same position. The mouth was dry, the hide clung close to the ribs, the temperature of the body 39.6°, pulse normal at 55, respiration 16, evacuation and urination decreased; all food refused. When driven from the stall nothing abnormal was noticeable except the peculiar position of the head and neck described above. As cases of cœnurus cerebralis occur often in the neighborhood, Giuseppe reached this diagnosis, although the visible symptoms did not fully correspond to those of that disease. On the advice of Giuseppe the animal was immediately killed. An examination of the brain yielded the following result: After the dura mater was removed the basilar portion of the pia mater was found sown over with translucent miliary tubercles. These were most thickly accumulated along the larger blood-channels and the groups of veins. Beyond this nothing worthy of note was found in the whole brain. It was simply a question of a probably primary tuberculous leptomeningitis basilar. I think that without the oft-recurring cases of cœnurus cerebralis, which led to a false diagnosis, one would have, at most, been compelled to assume some injury of the brain-matter as the cause, without attempting to define its position and nature, particularly as not a trace of tuberculosis could be detected in any of the internal organs.—*Idem.*

